# Targeting NRF2 Sensitizes Esophageal Adenocarcinoma Cells to Cisplatin through Induction of Ferroptosis and Apoptosis

**DOI:** 10.3390/antiox11101859

**Published:** 2022-09-21

**Authors:** Farah Ballout, Heng Lu, Zheng Chen, Tianling Hu, Lei Chen, Mary Kay Washington, Wael El-Rifai, Dunfa Peng

**Affiliations:** 1 Department of Surgery, University of Miami Miller School of Medicine, Miami, FL 33136, USA; 2 Sylvester Comprehensive Cancer Center, Miami, FL 33136, USA; 3 Department of Gastroenterology, Beijing Friendship Hospital, Capital Medical University, Beijing 100050, China; 4 Department of Pathology, Microbiology and Immunology, Vanderbilt University Medical Center, Nashville, TN 37232, USA; 5 Department of Veterans Affairs, Miami Healthcare System, Miami, FL 33136, USA

**Keywords:** esophageal adenocarcinoma, NRF2, Brusatol, lipid peroxidation, ferroptosis

## Abstract

Esophageal adenocarcinoma (EAC), the predominant type of esophageal cancer in the United States, develops through Barrett’s esophagus (BE)-dysplasia-carcinoma cascade. Gastroesophageal reflux disease, where acidic bile salts refluxate into the esophagus, is the main risk factor for the development of BE and its progression to EAC. The NFE2-related factor 2 (NRF2) is the master cellular antioxidant regulator. We detected high NRF2 protein levels in the EAC cell lines and primary tissues. Knockdown of NRF2 significantly enhanced acidic bile salt-induced oxidative stress, DNA damage, and inhibited EAC cell growth. Brusatol, an NRF2 inhibitor, significantly inhibited NRF2 transcriptional activity and downregulated the NRF2 target genes. We discovered that in addition to inducing apoptosis, Brusatol alone or in combination with cisplatin (CDDP) induced significant lipid peroxidation and ferroptosis, as evidenced by reduced xCT and GPX4 expression, two known ferroptosis markers. The combination of Brusatol and CDDP significantly inhibited EAC tumor xenograft growth in vivo and confirmed the in vitro data showing ferroptosis as an important mechanism in the tumors treated with Brusatol or Brusatol and CDDP combination. Our data support the role of NRF2 in protecting against stress-induced apoptosis and ferroptosis in EACs. Targeting NRF2 in combination with platinum therapy can be an effective strategy for eliminating cancer cells in EAC.

## 1. Introduction

Esophageal cancer remains the 7th most common and the 6th most lethal malignancy worldwide [1]. While the esophageal squamous carcinoma incidence is declining, the incidence of esophageal adenocarcinoma (EAC) has been rising rapidly during the past decades in the United States and Western countries, becoming the predominant type of esophageal cancer [2,3,4]. Barrett’s esophagus (BE), where the normal esophageal squamous epithelium is replaced by intestinal metaplastic columnar epithelium in the lower esophagus due to chronic gastroesophageal reflux disease (GERD), is one of the major risk factors [5,6]. BE can progress to EAC through the BE-dysplasia-carcinoma cascade [7,8]. Although new modalities for cancer treatments such as targeted therapy and immune therapy have been developed over the past decade, the prognosis of EAC patients remains poor, with an average 5-year survival rate below 20% [1,9,10]. There is an urgent need to develop novel therapeutic strategies for EAC to improve the clinical outcome.

In response to a GERD episode, which contains bile salts and an acidic gastric juice mixture (acidic bile salts), there is a significant increase in oxidative stress and subsequent DNA damage in esophageal epithelia [11,12,13]. Normal cells possess intact anti-oxidative systems that protect cells from oxidative stress and subsequent DNA damage and cell death [14]. However, during esophageal tumorigenesis, there is an impairment or dysfunction of several anti-oxidative enzymes such as glutathione S-transferases (GSTs) and glutathione peroxidases (GPXs) through epigenetic DNA hypermethylation [15], leading to high levels of oxidative stress with subsequent DNA damage [16,17]. Cancer cells are known to have higher oxidative stress levels than normal cells due to the activation of oncogenes, increased metabolic activities, and impaired mitochondrial functions [14]. Cancer cells must develop counteractive mechanisms to survive the lethal effects of oxidative stress. The NFE2-related factor 2 (NRF2) is the master regulator of cellular antioxidant properties that maintain cellular viability and homeostasis under stress conditions [14]. NRF2 is constitutively overexpressed in human cancers and is associated with almost all tumor hallmarks [18,19]. Therefore, targeting NRF2 has become an attractive area of research where several activators and inhibitors have been reported and tested in vitro and in vivo [20]. Brusatol is a degraded diterpenoid isolated from the Brucea javanica plant [21], which has been used in traditional Chinese medicine to treat various ailments. Brusatol has shown anti-NRF2 activity [22] by inhibiting its transcription network and suppressing cancer cell growth in vitro and in vivo [23]. In the present study, we investigated the role of NRF2 in Barrett’s related esophageal adenocarcinoma and determined the potential therapeutic efficacy of targeting NRF2 using Brusatol as a single agent and in combination with cisplatin.

## 2. Materials and Methods

### 2.1. Cell Lines

HEEC, a human normal esophageal squamous epithelial cell line, was purchased from ScienCell Research Laboratories (Carlsbad, CA, USA). BAR10T (kindly provided by Dr. Rhonda Souza), CPA, and CPB (purchased from American Type Culture Collection, ATCC, Manassas, VA, USA) are immortalized cell lines originating from Barrett’s esophagus without dysplasia (BAR10T, CPA) and with dysplasia (CPB). Five esophageal adenocarcinoma cell lines were used, FLO-1, ESO26, ESO51, OE19, and OE33 (purchased from MilliporeSigma, Burlington, MA, USA). HEEC cells were cultured in EpiCM-2 medium (ScienCell) with 5% FBS. BAR10T, CPA, and CPB were grown in Human Epithelial Cell Medium with the growth factor supplement (Cell Biologics, Chicago, IL, USA). FLO1 cells were cultured in Dulbecco’s modified Eagle’s medium (DMEM) supplemented with 10% FBS and antibiotics. ESO26, ESO51, OE19, and OE33 cells were cultured with RPMI 1640 medium supplemented with 10% FBS, antibiotics, and L-glutamine. All cell lines were grown at 37 °C in 5% carbon dioxide with mycoplasma checked every month using a Mycoplasma Detection Kit (SouthernBiotech, Birmingham, AL, USA). Cell line authentication is conducted routinely every six months through Labcorp’s Cell Line Authentication Services (Burlington, NC, USA).

### 2.2. Tissues Samples

Tissue microarrays (TMA) were obtained from the Tissue Pathology Core at Vanderbilt University Medical Center, Nashville, TN. These TMAs included de-identified archival tissues of normal esophagus (*n* = 82), Barrett’s esophagus (*n* = 49), high grade dysplasia (*n* = 14), and EACs (*n* = 146).

### 2.3. Reagents

Bile salts (BS) used included glycocholic acid (GCA), taurocholic acid (TCA), glycodeoxycholic acid (GDCA), glycochenodeoxycholic acid (GCDCA) and deoxycholic acid (DCA), which were purchased from MilliporeSigma. A cocktail of the five bile salts was prepared with an equimolar concentration of a mixture of each bile salt. We treated cells with 100 μM of BS cocktail (contains 20 μM of each BS) in acidic culture media (pH 4.0, acidic bile salts, ABS) for 10–20 min to mimic a GERD episode. The ABS cocktail reflected the bile acid mixture in the distal esophagus in patients with GERD [24]. The following antibodies were used in the study: NRF2 antibody (ABCAM, ab62352, Cambridge, MA, USA, used for WB), NRF2 antibody (Milliporesigma, ABE413, Burlington, MA, USA, used for IHC), HO1 antibody (Proteintech, 10701-1-AP, Rosemont, IL, USA, used for WB and IHC), β-actin antibody (Sigma-Aldrich, A5441, St. Louis, MO, USA, used for WB), caspase 3 (Cell Signaling, 9662, Danvers, MA, USA, used for WB) and cleaved caspase 3 (Cell Signaling, 9661, for WB and IHC), PARP (Cell Signaling, 9532, used for WB), cleaved-PARP (Cell Signaling, 5625, for WB), xCT/SLC7A11 (Cell Signaling, 12691, used for WB), xCT/SLC7A11 (Proteintech, 26864-1-AP, used for IHC), and Ki-67 antibody (Invitrogen, 14-5698-82, Waltham, MA, USA, used for IHC). NRF2 siRNAs were purchased from Dharmacon (M-003755-02-0005, Lafayette, CO, USA) and ThermoFisher Scientific (s9491, Waltham, MA, USA). Brusatol and cisplatin (CDDP) were purchased from MilliporeSigma.

### 2.4. Real-Time qRT-PCR

Total RNA was purified using the RNeasy Mini Kit (Qiagen, Valencia, CA, USA). Single-stranded complementary DNA was subsequently synthesized using the iScript cDNA Synthesis Kit (Bio-Rad, Hercules, CA, USA). The quantitative real-time reverse transcription (qRT-PCR) was carried out on an iCycler (Bio-Rad). Reactions were performed in triplicate; the threshold numbers were determined by iCycler software version 3.0 and were averaged. We used human HPRT1 gene as the internal reference gene for each sample and fold expression was calculated as previously reported [15], normalized to HPRT1. The DNA sequences of the primers used in the study are provided in Supplementary Table S1.

### 2.5. Colony Formation Assay

FLO-1 and OE33 cells were transfected with control siRNA or NRF2 specific siRNA using the LipoJet In Vitro Transfection Kit (SignaGen Laboratories, Frederick, MD, USA) according to the manufacturer’s protocols. Forty-eight hours after infection, cells were seeded in the density of 1000 cells/well in the 6-well plates and cultured at 37 °C for another two weeks. For the clonogenic survival assay, tumor cells were seeded in 6-well plates at the density of 1000 cells per well. The next day, the cells were treated with Brusatol (30 nM), CDDP (3 µM), or a combination of Brusatol (30 nM) and CDDP (3 µM) for 24 h, followed by the removal of the media and replacement of full media for two weeks. Each experiment was set in triplicate. Cells were stained with 0.5% crystal violet solution. The images of the plates were analyzed using ImageJ software (version 1.53k, National Institutes of Health, Bethesda, MD, USA) and statistically analyzed using Prism 9 software (version 9.3.1, GraphPad Software, San Diego, CA, USA).

### 2.6. Luciferase Reporter Assay

The ARE luciferase reporter assay was used to determine the NRF2 transcriptional activity, as previously reported [25]. Briefly, the cells were co-transfected with PGL 4.37 [luc2P/ARE/Hygro] reporter (Promega, Madison, WI, USA), together with renilla luciferase plasmid as the internal control using the PolyJet DNA transfection agent (SignaGen Laboratories). After 24 h transfection, the cells were treated with Brusatol at a concentration from 10 nM to 500 nM for 6 h. The cell lysates were prepared using 1X luciferase passive lysis buffer. Luciferase activity was measured using a dual-luciferase reporter assay system (Promega, Madison, WI, USA) following the manufacturer’s instructions in a FLUOstar OPTIMA microplate reader (BMG LABTECH, Cary, NC, USA). Luciferase activity was calculated by normalizing it to the corresponding renilla value and represented as relative luciferase activity.

### 2.7. Detection of Intracellular ROS Levels

Flow cytometry was used to determine the intracellular ROS levels using a CM-H2DCFDA dye (ThermoFisher), as previously described [16]. Briefly, cells were transfected with the control and NRF2 siRNA using the LipoJet regent. After 48 h of transfection, 1.5 × 10^5^ cells were seeded into 12-well plates. The next day, cells were treated with 100 μM ABS for 10 min, followed by washing with PBS and incubated with 5 μM CM-H2DCFDA for 30 min. Then, the cells were trypsinized and resuspended with 500 μL phenol red-free media. Cells were then subjected to flow cytometry analysis using a CytoFLEX Flow Cytometer (BECKMAN COULTER, Indianapolis, IN, USA).

### 2.8. CellTiter-Glo Cell Viability Assay

The CellTiter-Glo^®^ Luminescent Cell Viability Assay (Promega, Madison, WI, USA) was used to evaluate the cell viability after single or combination treatments, following the manufacturer’s instructions. In brief, cells were seeded in a 96-well plate at the density of 1500 cells/well. On the second day, cells were treated with Brusatol or cisplatin alone or with a combination of both at serial dilutions for three days. After three days, CellTiter-Glo^®^ Luminescent reagent was added on cells for 30 min incubation with shaking. The luminescence changes were measured using the FluolarStar microplate reader (BMG Labtech, Ortenberg, Germany). The dose–response curve and IC50 values were generated using GraphPad Prism 9 software.

### 2.9. Immunofluorescence Staining

Immunofluorescence staining assay was used to determine the DNA double strand break using antibody against γ-H2AX (p-H2AX, S139) and oxidative DNA damage using antibody against 8-oxoguanine as previous described [12,16]. Briefly, 1.2 × 10^4^ cells were seeded in an eight-well slide chamber. The next day, the cells were treated with ABS (100 μM) for 20 min, then recovered in complete media for 3 h after the removal of ABS. Cells were fixed with 4% paraformaldehyde for 45 min, followed by permeabilization for 10 min on ice. After blocking using the goat antiserum for 20 min, the cells were incubated with primary antibody against phosphor histone H2AX (Ser 139, Cell signaling) and 8-oxoguanine (1:100) (MAB3560, Sigma-Aldrich, St. Louis, MO, USA) overnight. The next day, the cells were incubated with Alexa Fluor 488 goat anti-rabbit and Alexa Fluor 568 goat anti-mouse (1:1000) secondary antibodies for 45 min. The slides were mounted with Vectashield mounting medium with DAPI (Vector Laboratories, Newark, CA, USA) and sealed with a coverslip. The images were captured by using the BZ-X710 KEYENCE All-in-one fluorescence microscope (Keyence Corporation of America, Itasca, IL, USA) and analyzed using ImageJ software (NIH).

### 2.10. Western Blot Assay

The Western blot assay was used to determine the protein levels of interest following the standard protocol. For the apoptosis assay, cells were treated with Brusatol or CDDP alone or their combination for 72 h. Cells were then harvested and lysed with the RIPA buffer (Santa Cruz Biotechnology, Dallas, TX, USA), sonicated, and centrifuged at 13,000 rpm for 10 min at 4 °C. The supernatant was collected, and the concentration of protein samples was determined using a Pierce BCA Protein Assay Kit (ThermoFisher). Protein samples were denatured in a 4X LDS sample buffer by heating at 90 °C for 10 min. The same amount of proteins were electrophoresed and transferred into the nitrocellulose membrane. The membrane was blocked with 5% milk for 1 h at room temperature and incubated with the primary antibodies overnight. The membrane was then washed thrice with the 1X TBST buffer and incubated with their corresponding secondary antibodies with HRP for 2 h. After washing thrice, the membranes were developed using the ECL kits and imaged using a Bio-rad ChemiDoc^TM^ XRS+ imager system (Bio-rad). The bands’ intensities were measured using the gel analysis tool of ImageJ software (NIH) and normalized to the intensity of the loading control, beta-actin.

### 2.11. Detection of Synergy Effect of Brusatol and Cisplatin (CDDP)

To explore whether there is a synergistic effect in inducing tumor cell death for Brusatol and CDDP, we analyzed the data using the SynergyFinder Plus online analysis tool (https://synergyfinder.org/#!/, accessed on 10 May 2022), following the instructions for experimental settings and analyses [26]. There are currently four major synergy models: highest single agent (HSA) [27], Loewe additivity (LOEWE) [28], Bliss independence (BLISS), and zero interaction potency (ZIP) [29]. The details of the methods and formulations of the models used in the analysis were reported earlier [26,30].

### 2.12. Detection of Lipid Peroxidation

The Click-iT™ Lipid Peroxidation Imaging Kit—Alexa Fluor™ 488 (ThermoFisher) was used to detect lipid peroxidation after the Brusatol and CDDP treatments. LAA (linoleamide alkyne) can incorporate into cellular membranes when incubating with cells. Upon lipid peroxidation, LAA is oxidized and produces 9- and 13-hydroperoxy-octadecadienoic acid (HPODE). These hydroperoxides decompose to multiple α and β-unsaturated aldehydes, which readily modify proteins at nucleophilic side chains. These alkyne-containing modified proteins can be subsequently detected using Click-iT™ chemistry for fixed cells. In brief, cells were seeded into an 8-well culture slide camber and cultured at 37 °C overnight. The next day, cells were added with Click-it LAA at a final concentration of 50 µM followed by treatment with Brusatol, CDDP, or both for 6 h in the presence of LAA. After treatments, cells were fixed in 1 mL of 3.7% formaldehyde in PBS for 15 min at room temperature. After washing of fixation, cells were added 1 mL of 0.5% Triton^®^ X-100 in PBS for 10 min at room temperature, followed by blocking in 1% BSA in PBS solution for 30 min. After blocking, cells were incubated with 0.5 mL of Click-iT^®^ reaction cocktail for 30 min at room temperature. Then, the cells were washed thrice and the slides were mounted with Vectashield mounting medium with DAPI. The images were captured by using a BZ-X710 KEYENCE All-in-one fluorescence microscope (Itasca, IL, USA). To confirm the results, we applied a flow cytometry assay using BODIPY™ 581/591 C11 dye (a lipid peroxidation sensor from ThermoFisher). Oxidation of the polyunsaturated butadienyl portion of the dye results in a shift in the fluorescence emission peak from ∼590 nm to ∼510 nm, which can be detected using flow cytometry. Cells were treated in a 6-well plate with Brusatol, CDDP, or both for 6 h. Cells were harvested and immediately subjected to flow cytometry analysis using the FITC and PE channels. The ratio of the signaling intensity of FITC/PE was shown.

### 2.13. In Vivo Treatments of Tumor Xenografts

Animal works were performed following the animal protocol (#UM 20-110) approved by the IACUC of the University of Miami. Animal care was in accordance with institutional guidelines. A total of 2 × 10^6^ OE33 cells were injected into SOD/SCID immune deficient mice (Jackson Laboratory, Bar Harbor, ME USA) on the flank subcutaneously [17]. Mice weight and tumor masses were monitored twice a week. Tumor volume was calculated using the formula: ½ length × width^2^. Drug delivery was not started until tumors reached approximately 200 mm^3^ (about 12 days after injection). Mice were randomly divided into four groups: control, PBS; Brusatol, 1 mg/kg, 3 times/week, IP; CDDP, 1 mg/kg, once a week, IP; Brusatol + CDDP, Brusatol, 1 mg/kg, 3 times/week, IP and CDDP, 1 mg/kg, once a week. Brusatol and CDDP were not given on the same day to minimize toxicity. After 30 days of treatments, the mice were euthanized and tumors were dissected and divided into two parts: one was snap frozen in liquid N_2_, the other was fixed in formalin solution for paraffin embedding and subsequent HE and IHC staining.

### 2.14. Immunohistochemistry

Immunohistochemistry staining was carried out using a Millipore Immunoperoxidase Secondary Detection System (MilliporeSigma). In brief, slides from the human tissue microarray or paraffin blocks of xenografting tumors were deparaffined in xylene. Antigen retrieval was performed by boiling the slides in pH 9 TE buffer for 12 min. Slides were incubated with 3% H_2_O_2_ for 10 min and blocking solution for 30 min. Slides were then incubated with primary antibodies overnight, followed by anti-mouse or anti-rabbit second antibodies and the ABC complex provided in the kit following the manufacturer’s instructions. For double immunohistochemistry staining of Ki-67 and cleaved caspase 3, the ImmPRESS Duet Double Staining Polymer Kit (Vector Laboratory, Burlingame, CA, USA) was used following the manufacturer’s instructions. The slides were evaluated under microscopy. The quantification of the staining intensity of xenografted tumor tissues was performed using ImageJ software from 10–20 randomly selected high magnification fields. For the tissue microarray, we evaluated the NRF2 expression levels as index scores 0–3 based on the staining intensity and frequency, as previously described [31].

### 2.15. Statistical Analysis

Biochemical experiments were performed in triplicate in at least two independent cell lines and conditions. Quantified results were expressed as the mean ± SD. All the statistical analyses were performed using Prism 9, version 9.3.1 (GraphPad). A *p* < 0.05 was considered statistically significant.

## 3. Results

### 3.1. NRF2 Is Constitutively Overexpressed in Esophageal Adenocarcinomas

**Figure 1:**
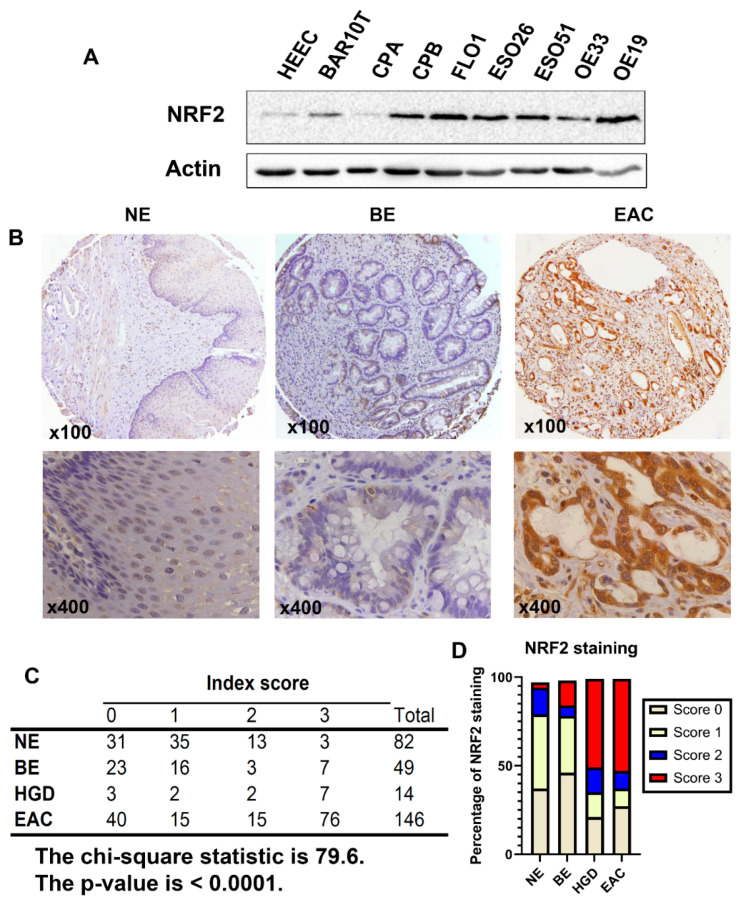
NRF2 protein expression is upregulated in high grade dysplasia and esophageal adenocarcinoma. (**A**) Western blotting analysis of NRF2 protein expression in cell lines originated from the normal esophageal squamous epithelium (HEEC), Barrett’s esophagus (BAR10T and CPA), dysplastic Barrett’s (CPB) and esophageal adenocarcinoma (FLO1, ESO26, ESO51, OE33, and OE19). (**B**) Immunohistochemistry analysis of the NRF2 protein expression in primary human tissues of normal esophagus (NE), Barrett’s esophagus (BE), and esophageal adenocarcinoma (EAC), showing overexpression in EAC. Representative images of 100× (upper panels) and 400× (lower panels) are shown. (**C**,**D**) A summary of NRF2 IHC staining scores in NE, BE, HGD, and EAC from the tissue microarrays, showing high levels of NRF2 in HGD and EAC tissues (the chi-square test, *p* < 0.0001).

NRF2 is overexpressed in several human malignancies [18,32,33]. To determine whether NRF2 is overexpressed in EAC, we performed Western blotting analysis of cell lines from esophageal cells. As shown in Figure 1A, the NRF2 protein levels were higher in Barrett’s dysplastic cell line, CPB, and all EAC cell lines than in the normal esophageal squamous cells (HEEC) and non-dysplastic Barrett’s esophagus cells (BAR10T and CPA). Immunohistochemistry confirmed that the NRF2 protein levels were significantly higher in HGD (high grade dysplasia) and EAC tissues than that in the normal esophagus (NE) and Barrett’s esophagus (BE) tissues, where the majority (>60%) of the primary HGD/EAC tissue samples scored 2–3 (Figure 1B–D).

### 3.2. NRF2 Protects against Reflux-Induced Oxidative Stress and DNA Damage in EAC

**Figure 2:**
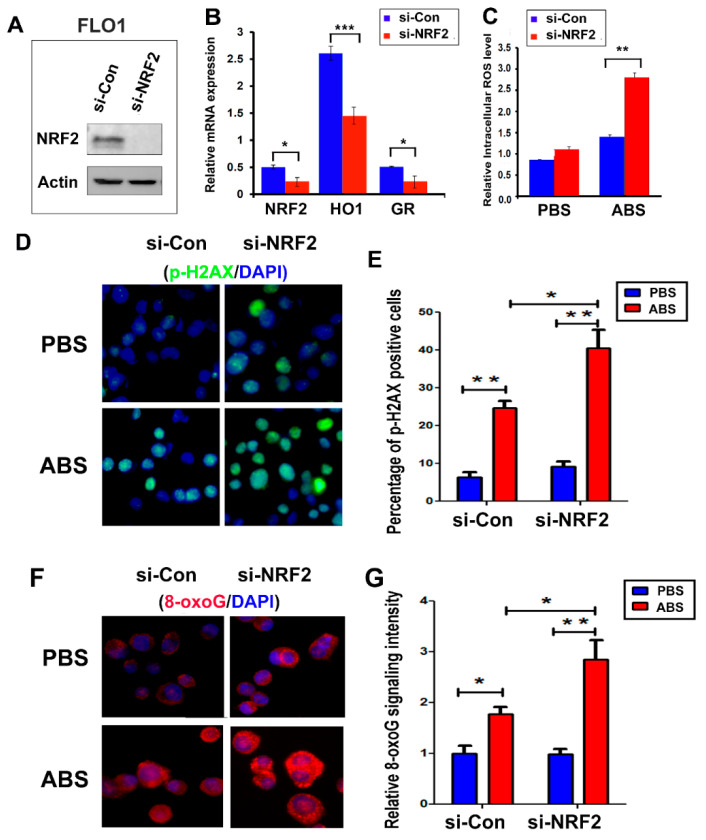
Knockdown of NRF2 expression in EAC cells promoted tumors to ABS-induced double strand break and oxidative DNA damage. (**A**) Western blotting shows the NRF2 level after the knockdown of NRF2 using an NRF2 siRNA in FLO1 cells. (**B**) Real-time RT-PCR shows the downregulation of NRF2 and its downstream target genes, HO1 and GR. (**C**) Knockdown of NRF2 sensitized cells to ABS-induced oxidative stress, indicated as significantly increased intracellular ROS level. (**D**,**E**) Immunofluorescence staining of γ-H2AX (p-H2AX, S139), a double strand break marker, indicating that knockdown of NRF2 promoted ABS-induced double strand break. (**F**,**G**) Immunofluorescence staining of 8-oxoguanine (8-oxoG), an oxidative DNA damage marker, indicating that knockdown of NRF2 significantly promoted the ABS-induced oxidative DNA damage level. * *p* < 0.05; ** *p* < 0.01; *** *p* < 0.001.

EAC cells are constantly exposed to acidic bile salts (ABS) under GERD conditions. ABS exposure induces significant ROS and oxidative stress. Accumulation of ROS is an early event that occurs within minutes, followed by subsequent changes in oxidative DNA damage and double stranded DNA damage. The accumulation of DNA damage in esophageal cells results in cell death [11,16]. Based on the biological sequence of events and our experimental optimization, we utilized early time points to measure ROS, whereas later timepoints were chosen to detect changes in DNA damage and cell death. To determine whether the observed high expression of NRF2 protects EAC cells from ABS-induced oxidative stress and DNA damage under conditions of GERD, we knocked down NRF2 protein using NRF2 specific siRNAs (Figure 2A). For additional confirmation, we also measured the expression of two NRF2 transcription targets, *HO1* and *GR* (Figure 2B). As expected, the knockdown of NRF2 enhanced ABS-induced oxidative stress (Figure 2C). NRF2 knockdown sensitized EAC cells to the ABS-induced double strand breaks, as evidenced by increased γH2AX, a known double strand break marker (Figure 2D,E and Figure S1C) and oxidative DNA damage that was represented by increased 8-oxoguanine (8-oxoG) [34] (Figure 2F,G and Figure S1D). Similarly, NRF2 knockdown enhanced cisplatin (CDDP)-induced oxidative stress (Figure S2C,D) and sensitized EAC cells to CDDP-induced double strand breaks, as shown by increased γH2AX (Figure S2E,F).

### 3.3. NRF2 Knockdown Inhibits EAC Cell Growth In Vitro

**Figure 3:**
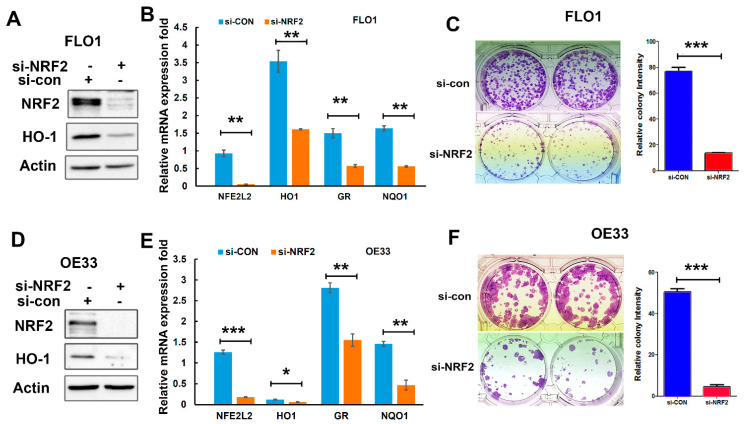
Knockdown of NRF2 inhibited EAC cell growth in vitro. (**A**,**D**) Western blotting indicates protein levels of NRF2 and HO1 (an NRF2 target gene) after NRF2 knockdown using an NRF2 siRNA in FLO1 (**A**) and OE33 (**D**) cells. (**B**,**E**) Real-time qPCR shows the downregulation of NRF2 target genes (HO1, GR, and NQO1) in FLO1 (**B**) and OE33 (**E**) cells after NRF2 knockdown. (**C**,**F**) The colony formation assay demonstrated significantly fewer and smaller colonies after NRF2 knockdown in FLO1 cells (**C**) and OE33 cells (**F**). * *p* < 0.05; ** *p* < 0.01; *** *p* < 0.001.

To determine the role of NRF2 on EAC cell survival and growth, we performed a colony formation assay following NRF2 knockdown in EAC cells. As shown in Figure 3, NRF2 knockdown significantly decreased the gene expression of its target genes (Figure 3A,B,D,E) and suppressed the tumor cell growth in both FLO1 (Figure 3C) and OE33 cells (Figure 3F).

### 3.4. Brusatol Inhibits NRF2 Activity and Induces EAC Cells’ Death

**Figure 4:**
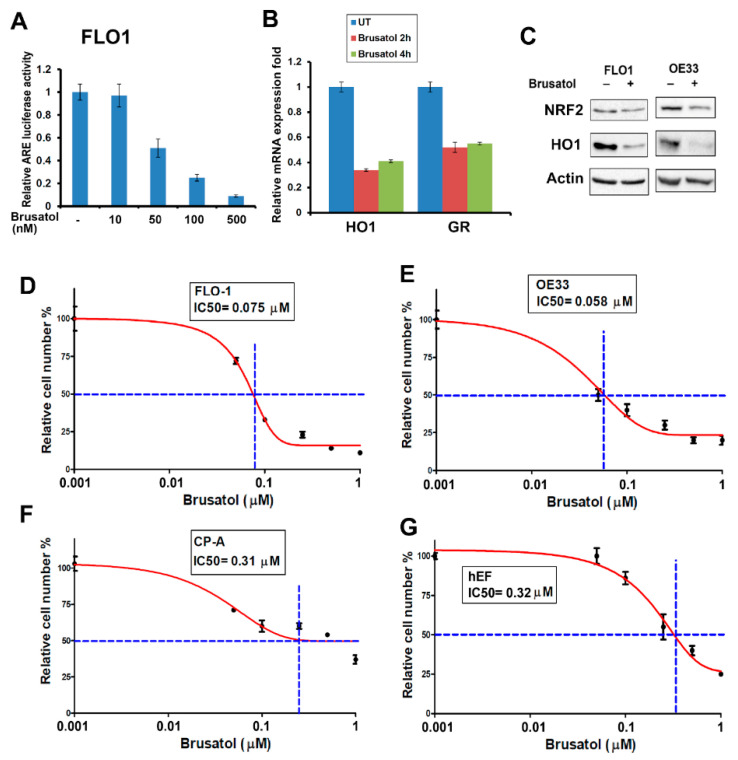
Brusatol inhibited NRF2 activity and killed EAC cells at low concentrations. (**A**) The ARE (antioxidant response element) luciferase reporter assay indicates the inhibition of NRF2 ARE activity at a 50–100 nM concentration of Brusatol. (**B**) Real-time RT-PCR shows the downregulation of NRF2 downstream target genes, HO1 and GR after Brusatol treatment. (**C**) Western blot displays the downregulation of the HO1 protein level after Brusatol treatment in the EAC cells. (**D**–**G**) The CellTiter Glo cell viability assay indicates that EAC cells were sensitive to Brusatol treatment with an IC50 below 100 nM (**D**,**E**), whereas the Barrett’s esophagus cells (**F**) and normal esophageal fibroblast cells (**G**) were more resistant to Brusatol treatment. The blue dot lines in D-G indicate the crosspoints between the 50% survival line and the dose-response curve.

Brusatol is a diterpenoid isolated from the Brucea javanica plant [21], which has been shown to inhibit NRF2 activity. We confirmed the inhibitory effect of Brusatol (at 50–100 nM) on NRF2 transcriptional activity using the ARE luciferase reporter assay (Figure 4A). We validated the effect of Brusatol, showing a downregulated expression of the NRF2 downstream target genes, *HO1* and *GR* (Figure 4B,C). The ATPglo assay demonstrated Brusatol’s IC50 at 50–100 nM in the EAC cell lines (Figure 4D,E), whereas normal esophageal fibroblast cells (hEF) and non-dysplastic Barrett’s cells (CP-A) were relatively resistant to Brusatol (Figure 4F,G). Of note, other NRF2 inhibitors such as ML385 [35] and AEM1 [36] were less effective than Brusatol in our models and failed to reduce the activity of NRF2 and the expression HO1 at lower doses (data not shown).

### 3.5. Brusatol Synergizes with Cisplatin in Inducing EAC Cells’ Death

**Figure 5:**
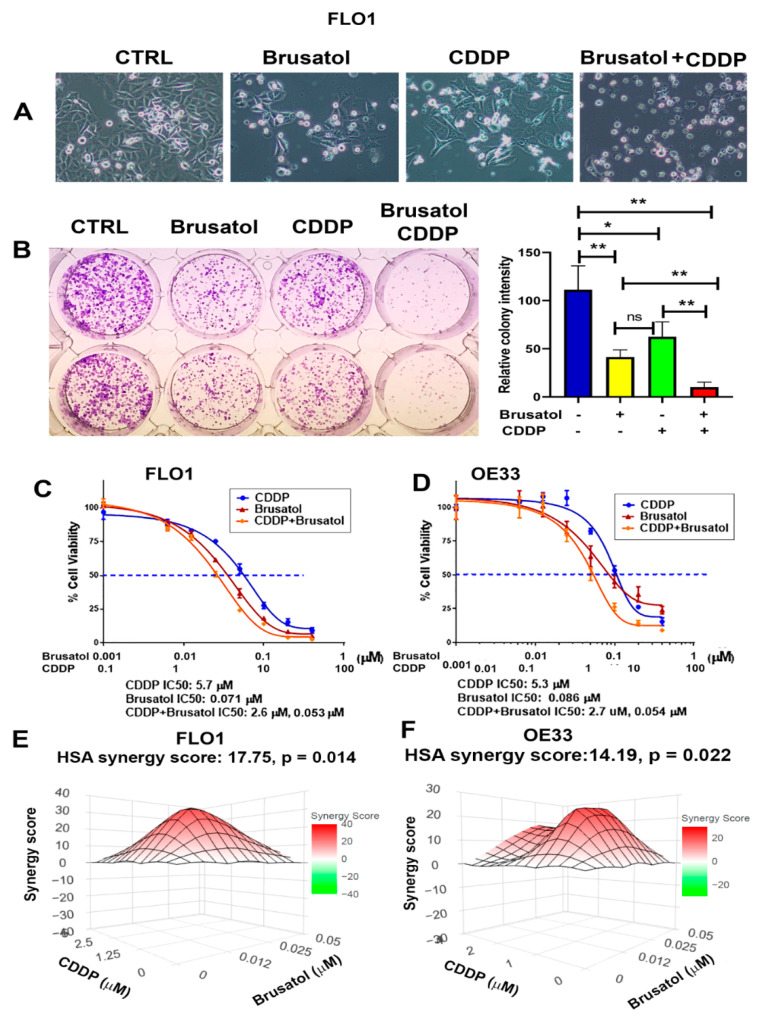
Brusatol synergized with cisplatin (CDDP) in killing EAC cells. (**A**) Bright images of cells treated with Brusatol, CDDP, or both for 3 days. (**B**) Clonogenic survival assay shows that combination treatment of Brusatol and CDDP killed a significantly more tumor cells. (**C**,**D**) The CellTiter Glo cell viability assay using Brusatol, CDDP, and both in FLO1 (**C**) and OE33 (**D**) cells. The IC50 of CDDP and Brusatol were plotted using Prism software. Data show that the combination treatment led to a significant 50% drop in CDDP IC50. (**E**,**F**) Synergy analysis using online synergyfinder tool (http://synergyfinder.org/, accessed on 10 May 2022) in FLO1 (**E**) and OE33 cells (**F**), demonstrating that a significant synergy effect occurred when applying Brusatol and CDDP. * *p* < 0.05; ** *p* < 0.01; ns, not significance.

CDDP is a common chemotherapeutic agent in the treatment of EAC patients [37]. However, the presence of advert toxicity and subsequent development of chemoresistance is a big problem. Therefore, to see whether Brusatol has a synergistic effect with CDDP, we treated EAC cells with Brusatol, CDDP alone, or a combination of both. As shown in Figure 5, the combination treatment of Brusatol and CDDP generated significantly more tumor cell death than CDDP or Brusatol alone (Figure 5A–D and Figure S3). In addition, the IC50 values for CDDP and Brusatol were significantly reduced when the combination treatment was applied (Figure 5C,D). Of note, the combination treatment worked well in the EAC cells with intrinsic (SKGT4 and OE19 cells) and secondary (FLO1 CDDP-R cells) resistance to CDDP (Figure S4). We also carried out synergy analyses using an online synergyfinder tool (http://synergyfinder.org/, accessed on 10 May 2022 ) [26]. We detected a significant synergistic effect of the combination of CDDP and Brusatol in both the FLO1 and OE33 cell lines (Figure 5E,F).

### 3.6. Brusatol and Its Combination with CDDP Induce Ferroptosis in EAC Cells

**Figure 6:**
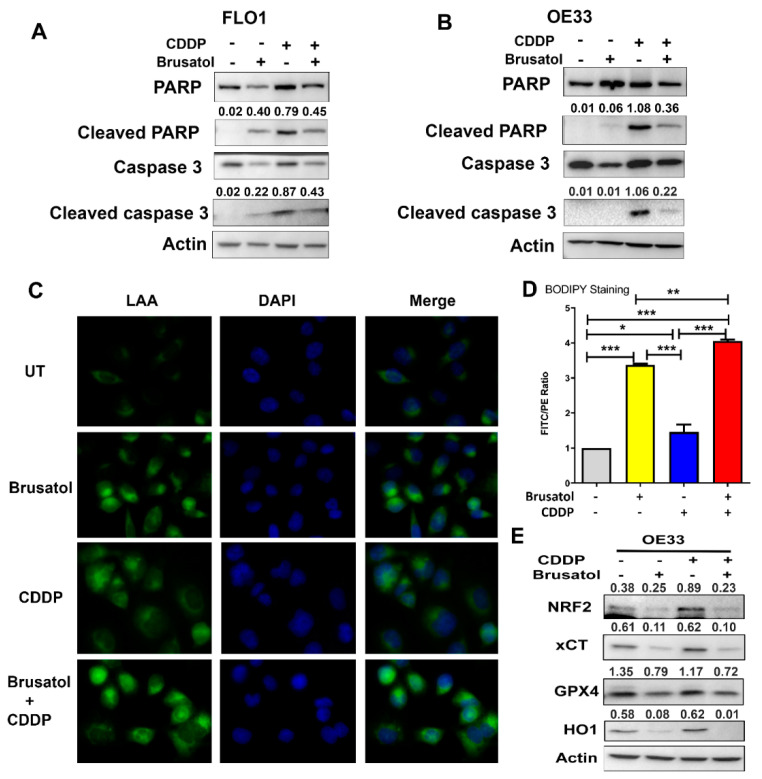
Brusatol treatment induced significant ferroptosis in addition to apoptosis. (**A**,**B**) Western blotting analyses of apoptotic markers, cleaved PARP, and cleaved caspase 3 in FLO1 (**A**) and OE33 (**B**) cells. Data show that although Brusatol and CDDP combination induced much more cell death, the two apoptotic markers were lower than that in cells treated with CDDP alone, suggesting that other types of cell death occurred, in addition to apoptosis. (**C**) The LAA lipid peroxidation assay displays the level of lipid peroxidation (green signaling). (**D**) A summary graph of the flow cytometry results using BODIPY^®^ 581/591 C11 (a lipid peroxidation sensor), confirming the significant peroxidation levels induced by Brusatol alone and Brusatol in combination with CDDP. (**E**) Western blotting analyses of two known ferroptosis markers, xCT and GPX4, validated the ferroptosis induced by Brusatol or Brusatol in combination with CDDP. The numbers above each band in (**A**,**B**,**E**) indicate the relative band intensity as normalized to the intensity of the loading control, Actin. * *p* < 0.05; ** *p* < 0.01; *** *p* < 0.001.

Apoptosis is a major mechanism by which most chemotherapeutic agents work to induce cancer cell death. Our findings demonstrated the induction of apoptosis in response to treatments, as expected (Figure 6A,B). Interestingly, we observed more cancer cell death with combination treatment (Figure 5), which was not reflected by the changes in the levels of cleaved PARP and cleaved caspase 3, the two known apoptosis markers (Figure 6A,B). These data suggested that in addition to apoptosis, another type of cancer cell death was induced by the combination treatment. NRF2 is the master antioxidant transcription factor regulating key physiological and metabolic activities including lipid peroxidation [14,38]. Brusatol, as an NRF2 inhibitor, is expected to result in high oxidative stress levels. We hypothesized that this effect would lead to lipid peroxidation with subsequent ferroptosis, contributing to the observed high levels of EAC cell death. To confirm this, we applied several assays to detect lipid peroxidation after Brusatol and CDDP treatments. As shown in Figure 6C, the linoleamide alkyne (LAA) lipid peroxidation assay (The Click-iT™ Lipid Peroxidation Imaging Kit) demonstrated that LAA lipid peroxidation was observed in cells treated with Brusatol, with the highest signaling in cells receiving a combination treatment; a mild lipid peroxidation was also observed in the CDDP-treated cells. Similar results were observed in other EAC cell lines (Figure S5). We validated the results by flow cytometry analysis using the BODIPY™ 581/591 C11 reagent (a lipid peroxidation sensor) (Figure 6D). Western blotting analysis further confirmed the induction of ferroptosis in Brusatol treated cells (alone or in combination with CDDP), as indicated by significantly decreased levels of xCT and GPX4, two known ferroptosis markers (Figure 6E and Figure S6). To validate the results, we applied a ferroptosis inhibitor, ferrostatin 1. The administration of ferrostatin 1, together with Brusatol and CDDP, protected cells from cell death (Figure S7).

### 3.7. Brusatol and CDDP Combination Are Synergistic In Vivo

**Figure 7:**
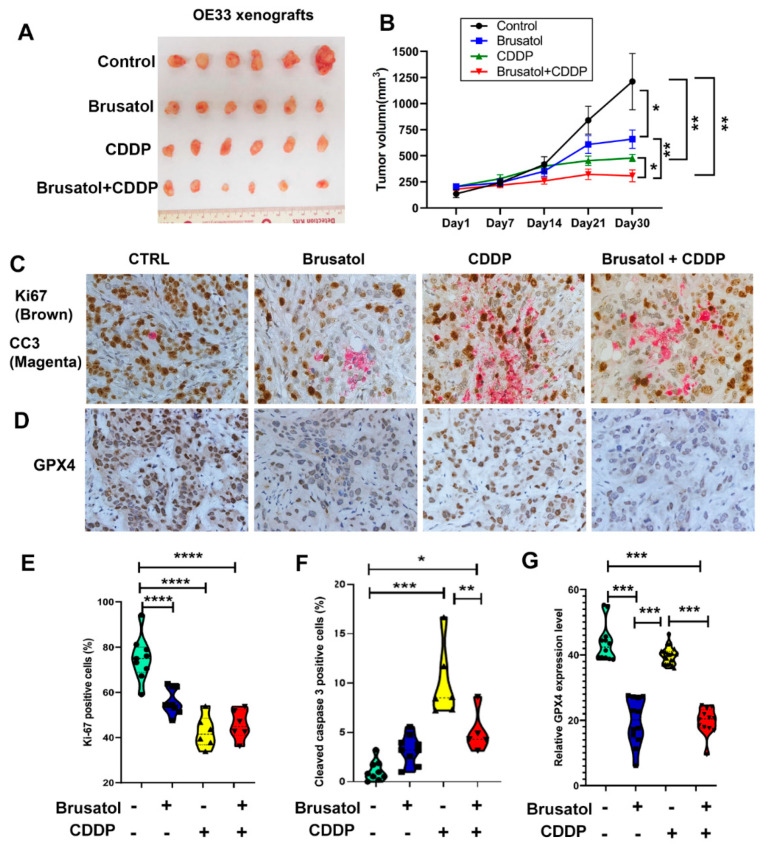
Brusatol synergized with CDDP in killing tumor cells in vivo. (**A**) Representative images of xenogrfting tumors after 30 days of treatments of Brusatol, CDDP, or Brusatol + CDDP. (**B**) The tumor growth curve shows that Brusatol and CDDP alone significantly slowed tumor growth, but the Brusatol and CDDP combination generated the most suppressive effect. (**C**) Dual immunohistochemistry staining of the cell proliferation marker (Ki-67, brown) and apoptosis marker (cleaved caspase 3, magenta) in the xenografted tumor tissues. The quantitative data using ImageJ software are shown in (**E**) (Ki-67) and (**F**) (cleaved caspase 3). (**D**) Immunohistochemistry staining of ferroptosis marker, GPX4, in xenografted tumor tissues shows the significant downregulation of GPX4. The quantitative data using ImageJ software are shown in (**G**). *, *p* < 0.05; **, *p* < 0.01; ***, *p* < 0.001; ****, *p* < 0.0001.

To test the effect of Brusatol on tumor growth in vivo, we established an EAC tumor xenografting model. OE33 cells were xenografted into SCID/NOD immune deficient mice subcutaneously. Brusatol, CDDP, or a combination of both, was administered through intraperitoneal (IP) injection when tumor volumes reached approximately 200 mm^3^, as described in the Methods section. As shown in Figure 7A,B, the administration of Brusatol or CDDP as a single agent slowed down tumor growth compared to the control group. Treatment with a combination of Brusatol and CDDP resulted in significantly greater inhibition of tumor growth than single agents. Immunohistochemistry staining of xenografted tumors demonstrated reduced expression levels of NRF2 and HO1 in tumors treated with Brusatol alone and Brusatol in combination with CDDP (Figure S8A,B,E,F). Immunohistochemistry analyses of tumor cell proliferation (Ki-67) and apoptosis (cleaved caspase 3, CC3) showed a reduction in proliferating cells (Figure 7C,E) along with higher cleaved caspase 3 rates in all of the treatment groups (Figure 7C,F). In agreement with the in vitro results, we observed a higher number of apoptotic cells in the CDDP-treated tumors than in tumors treated with the combination (Figure 7F). In contrast, immunohistochemistry analyses of ferroptosis markers GPX4 and xCT demonstrated significantly lower levels of GPX4 (Figure 7B,G) and xCT (Figure S8C,G) in tumors treated with Brusatol alone and in combination, consistent with the in vitro data.

## 4. Discussion

The incidence of esophageal adenocarcinoma (EAC) has been rising rapidly during the past few decades in the United States and Western countries, becoming the predominant type of esophageal cancer [39,40]. EACs develop through gastroesophageal reflux disease (GERD)-Barrett’s esophagus (BE)-dysplasia-carcinoma cascades [40,41,42]. It is known that acidic bile salt (ABS) exposure during this process generates significant oxidative stress and DNA damage levels in vitro and in vivo [11,25,43,44,45]. Tumor cells must develop protective mechanisms to survive in this harsh microenvironment. The NFE2-related factor 2 (NRF2) is the master regulator of cellular antioxidant properties that play crucial roles in maintaining cellular homeostasis [46,47,48]. Transient induction of NRF2 expression is an important step for protection against acidic bile acid-induced oxidative stress and DNA damage in normal and BE cells [25]. Here, we showed that NRF2 is constitutively overexpressed in HGD/EAC cells, providing a survival advantage. High levels of NRF2 allow neoplastic cells to evade the lethal effects of high ROS levels imposed by chronic inflammation or chemotherapy. NRF2 plays dual functions in tumorigenesis [49,50,51]. On one hand, NRF2 protects normal cells from stress-induced DNA damage, which may contribute to tumorigenesis if not repaired properly and promptly. On the other hand, tumorigenic cells are addicted to high levels of NRF2 for their survival and progression, known as the dark side of NRF2 [20].

Abnormal expression of NRF2 is involved in almost all the hallmarks associated with cancer progression, metastasis, and drug resistance [18,19,50,52,53]. Because cancer cells are addicted to high levels of NRF2 [32,33], the premise of NRF2 targeting has become an attractive idea [54]. Brusatol, a derivative from the Brucea javanica plant [21], can reduce NRF2 protein and activity levels through enhanced ubiquitination and degradation of NRF2 [22]. Brusatol has shown promising results in in vitro and in vivo cancer models [23,55]. In this study, we tested Brusatol alone and in combination with cisplatin (CDDP), a common drug for the treatment of EAC. While Brusatol alone was effective in vitro and in vivo, its combination with CDDP was synergistic and highly efficacious in eliminating cancer cells. Our data indicated that Brusatol or CDDP as single agents inhibited tumor growth. The combination of Brusatol and CDDP was synergetic at half the doses reported in the literature [22,23,55]. Our data suggest that this combination approach can be a therapeutic strategy for EAC patients, achieving tumor suppression with possibly lower toxicity. Mechanistically, treatments with Brusatol, CDDP alone, or a combination of both induced apoptosis, as expected. We found that the combination induced more cancer cell death through the induction of ferroptosis. The occurrence of ferroptosis was largely dependent on NRF2 inhibition by Brusatol. Ferroptosis, a form of cell death, is recognized as an important molecular mechanism that can effectively kill cancer cells [56,57]. Glutathione peroxidase 4 (GPX4) is a key inhibitor of ferroptosis, and its downregulation in response to therapy coincides with the occurrence of ferroptosis [58,59]. The reduced levels of xCT and GPX4 following treatment with Brusatol support the occurrence of ferroptotic cancer cell death [60,61], which was mitigated following treatment with a ferroptosis inhibitor, ferrostatin 1. As NRF2 plays an important role in mitigating lipid peroxidation [14,38], we hypothesized that ferroptosis was associated with an increase in lipid peroxidation following NRF2 inhibition. Indeed, our results demonstrated a significant increase in lipid peroxidation and ferroptosis by Brusatol. On the other hand, while CDDP induced high levels of apoptosis, it had minimal effects on lipid peroxidation and ferroptosis.

Although Brusatol has been reported as an NRF2 inhibitor and was used for several in vitro and in vivo studies [22,23,62], recent reports suggest off-target effects [63]. At higher concentrations, Brusatol can inhibit global protein synthesis [63] and incur off-target effects and excessive toxicity. In our experiments, we utilized relatively low doses and demonstrated the inhibition of ARE reporter and reduced expression of HO-1, a classical target of NRF2. Nevertheless, there is an urgent need to develop more specific NRF2 inhibitors for future investigations.

## 5. Conclusions

In summary, our data indicate that the overexpression of NRF2 in EAC promotes tumor survival. Targeting NRF2 synergizes with cisplatin through inducing significant lipid peroxidation and ferroptosis.

## Data Availability

All the data are contained within the article and the Supplementary Materials.

## Supplementary Materials

The following supporting information can be downloaded at: https://www.mdpi.com/article/10.3390/antiox11101859/s1, Figure S1. Knockdown of NRF2 enhanced ABS-induced DNA damage in OE33 cells. Figure S2. Knockdown of NRF2 enhanced CDDP-induced oxidative stress and DNA damage. Figure S3. Combination treatment of Brusatol and CDDP generated more cell death than CDDP and Brusatol alone. Figure S4. Combination treatment of Brusatol and CDDP generated significantly more tumor cell death than CDDP and Brusatol alone in the CDDP resistant cells. Figure S5. The LAA lipid peroxidation assay in the FLO1 and SKGT4 cells. Figure S6. Brusatol or a combination of Brusatol and CDDP induced ferroptosis in the FLO1 and OE19 cells. Figure S7. Ferrostatin 1 protected cells from cell death. Figure S8. The IHC staining of NRF2, HO1, xCT in xenografting tumor tissues. Table S1. Primers for real-time PCR.
