# Supplementary material for: Targeting NRF2 Sensitizes Esophageal Adenocarcinoma Cells to Cisplatin through Induction of Ferroptosis and Apoptosis
Source: Antioxidants (Basel). 2022 Sep 21;11(10):1859. doi: 10.3390/antiox11101859 (PMC9598457; doi:10.3390/antiox11101859)
Supplement: Supplementary file 1 [file antioxidants-11-01859-s001.zip › antioxidants-1864262-supplementary.pdf]

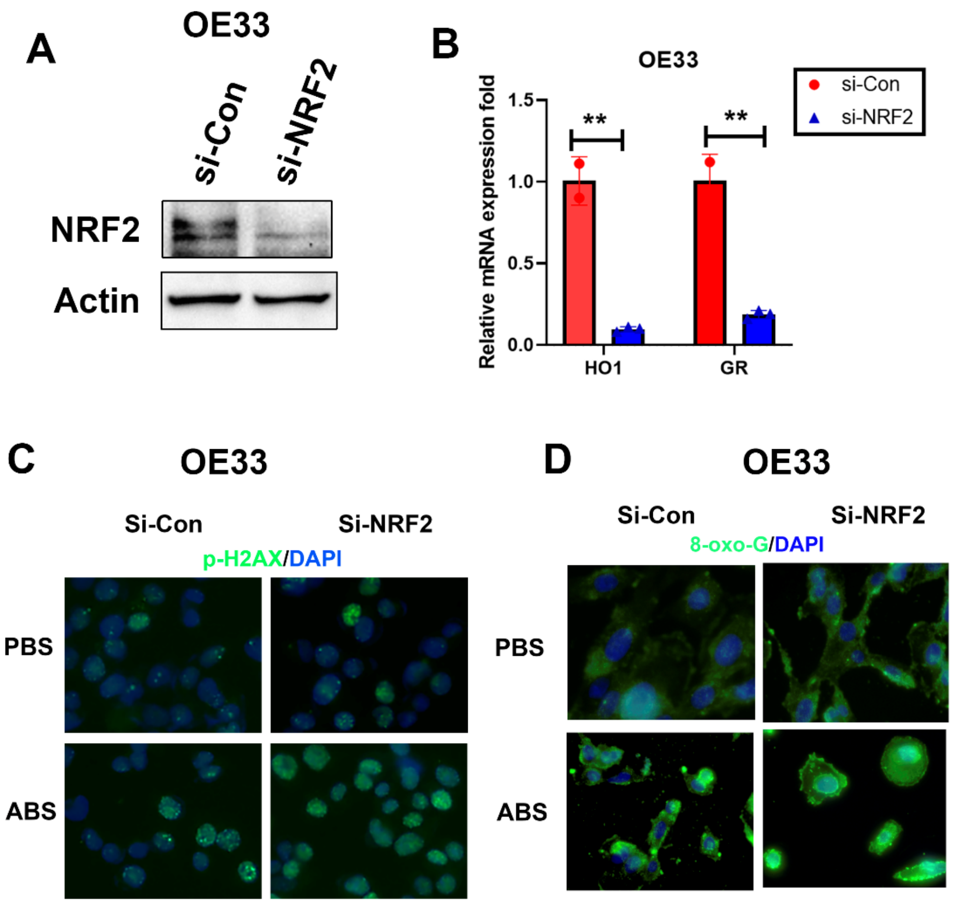

***Supplementary Figure S1. Knockdown of NRF2 enhanced acidic bile salts (ABS)-induced DNA damage**. **A**, western blotting demonstrated success knockdown of NRF2 by a NRF2 siRNAin OE33 cells. **B**, qRT-PCR showed downregulation of known NRF2 target genes after knockdown of NRF2. **C**, immunofluorescence staining of p-H2AX (γH2AX) for double stand break after ABS treatment. **D,** immunofluorescence staining of 8-oxoguanine (8-oxoG), an oxidative DNA damage marker.*

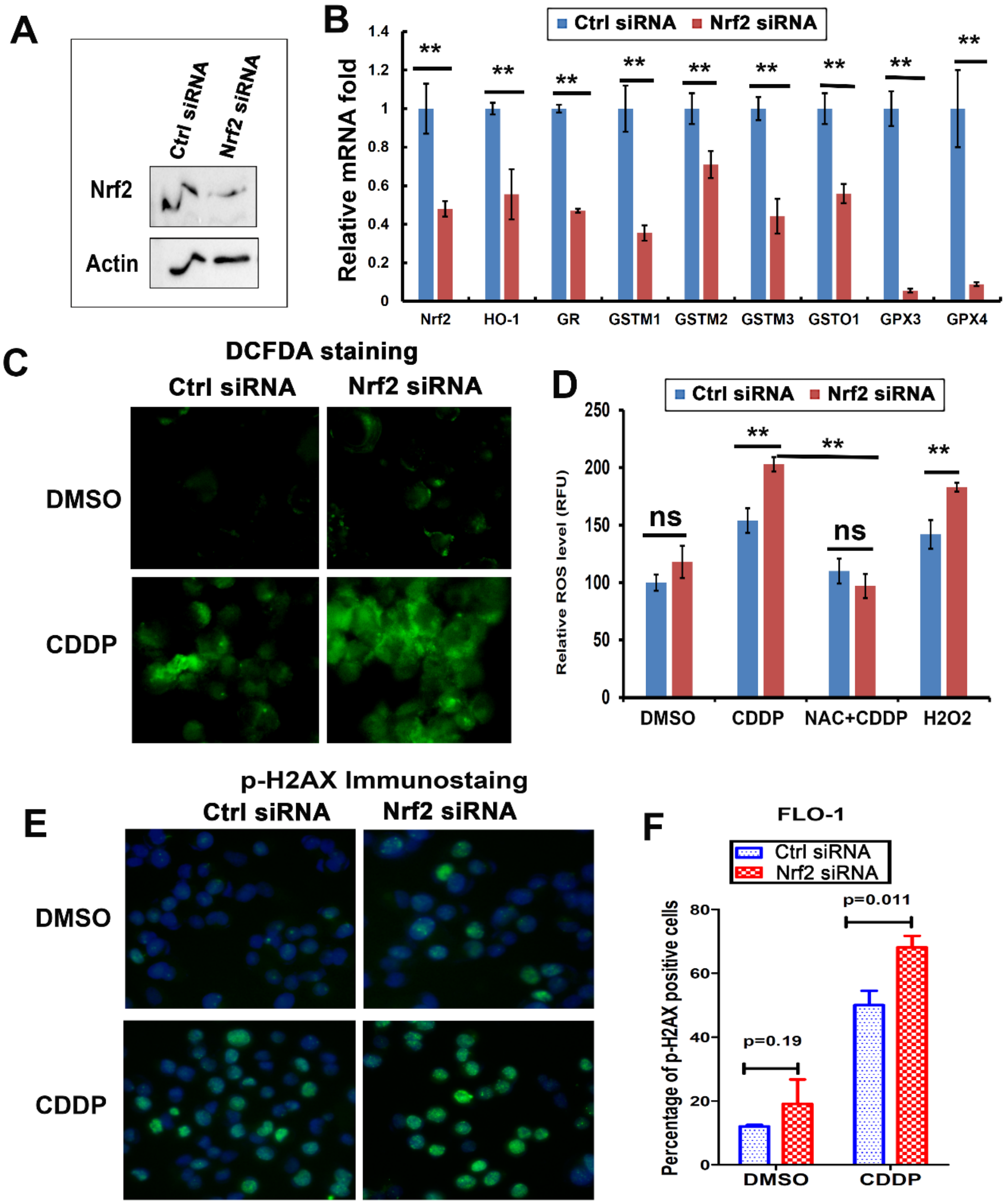

Supplementary Figure S2. Knockdown of NRF2 enhanced CDDP-induced oxidative stress and DNA damage. ***A***, *western blotting demonstrated success knockdown of NRF2 by a NRF2 siRNA in FLO1 cells.* ***B***, *qRT-PCR showed downregulation of multiple known NRF2 target genes after knockdown of NRF2.* ***C***, *DCFDA staining for intracellular ROS level after cisplatin (CDDP) treatment in NRF2 siRNA and control cells.* ***D***, *Bar graph summarizes the relative intracellular ROS levels detected using DCFDA. NAC is ROS scavenger and H2O2 as positive control.* ***E***, *immunofluorescence staining of p-H2AX (γH2AX) for double stand break after CDDP treatment.* ***F***, *Quantification of γH2AX positive cells.*

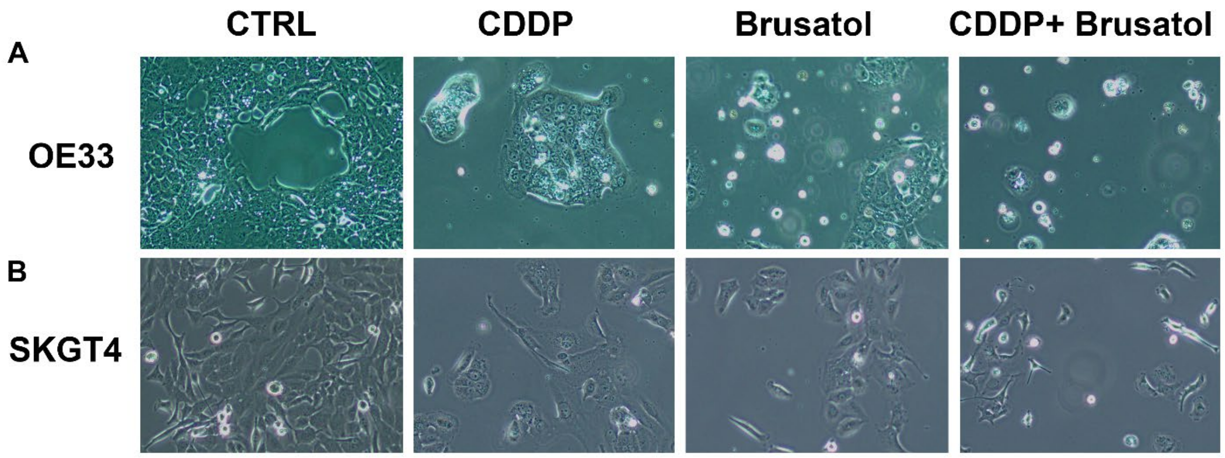

***Supplementary Figure S3. Combination treatment of Brusatol and CDDP generated more cell death than CDDP and Brusatol alone**. OE33 cells (**A**) and SKGT4 cells (**B**) were treated with Brusatol and CDDP alone or combination for 72h. Images were taken using an inverted microscope.*

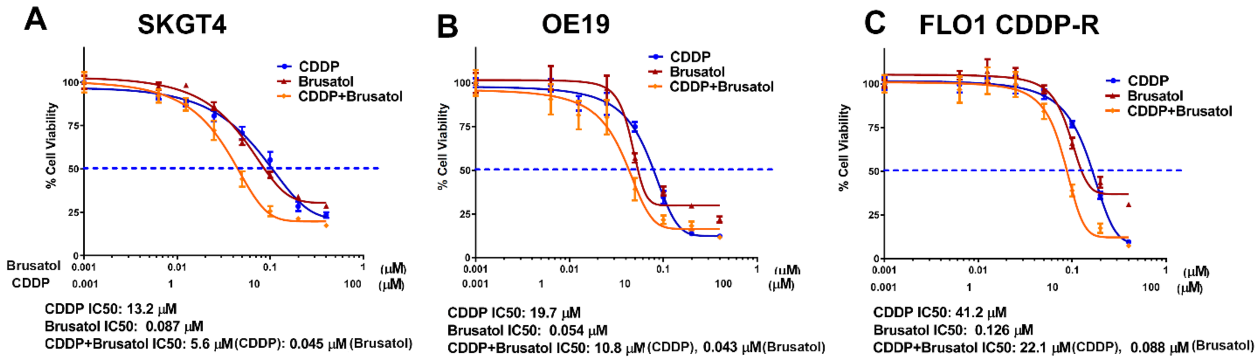

***Supplementary Figure S4. Combination treatment of Brusatol and CDDP generated significant more tumor cell death than CDDP and Brusatol alone in CDDP resistant cells**. SKGT4 (**A**), OE19 (**B**), two EAC cell lines resistant to CDDP and FLO1 CDDP-R (**C**, a FLO1 subline resistant to CDDP) were treated with Brusatol and CDDP alone or combination in a series deducted concentrations. Cell viability was measured 3 days after treatments using CellTiter Viability Assay. Results were analyzed using Prism software and were presented as IC50. Data show that Brusatol reduced about 50% CDDP IC50 in these CDDP resistant cells.*

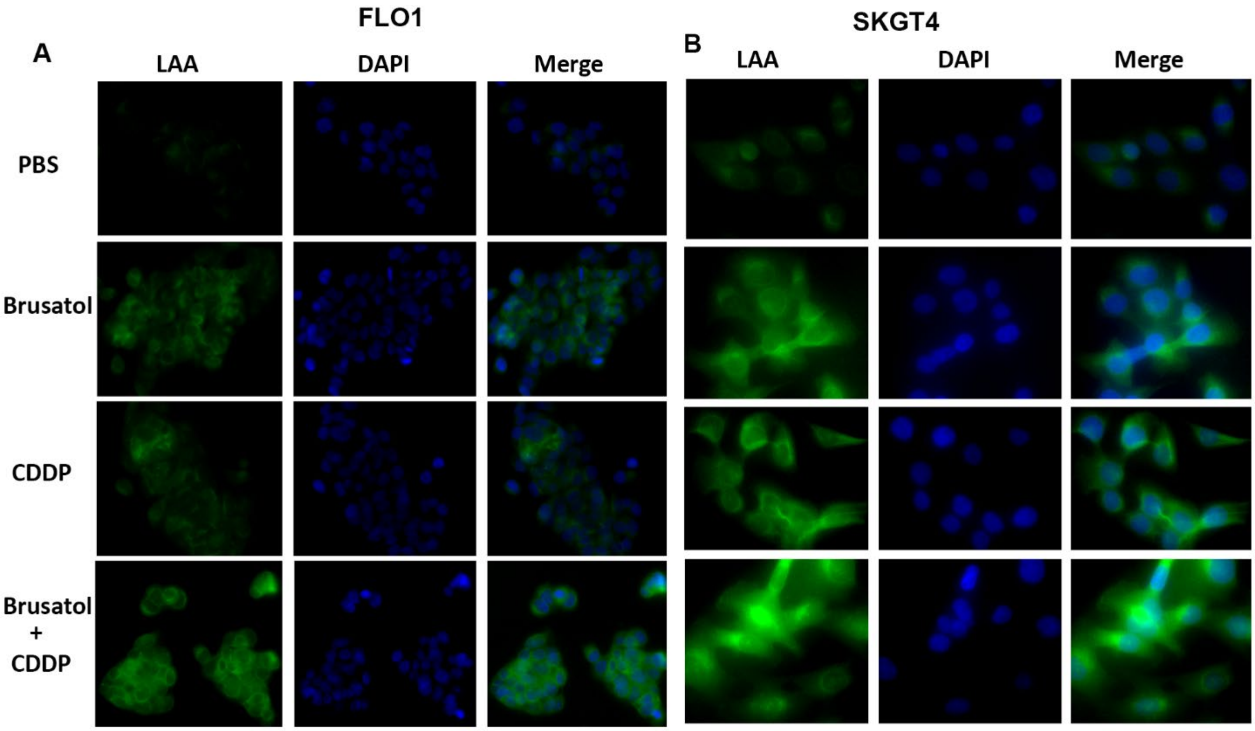

Supplementary Figure S5. LAA lipid peroxidation assay in FLO1 and SKGT4 cells*. LAA was co-incubated with Brusatol or CDDP or both for 6h, then following manufactory's protocol described in Method section. The lipid peroxidation levels were illustrated as green signaling.*

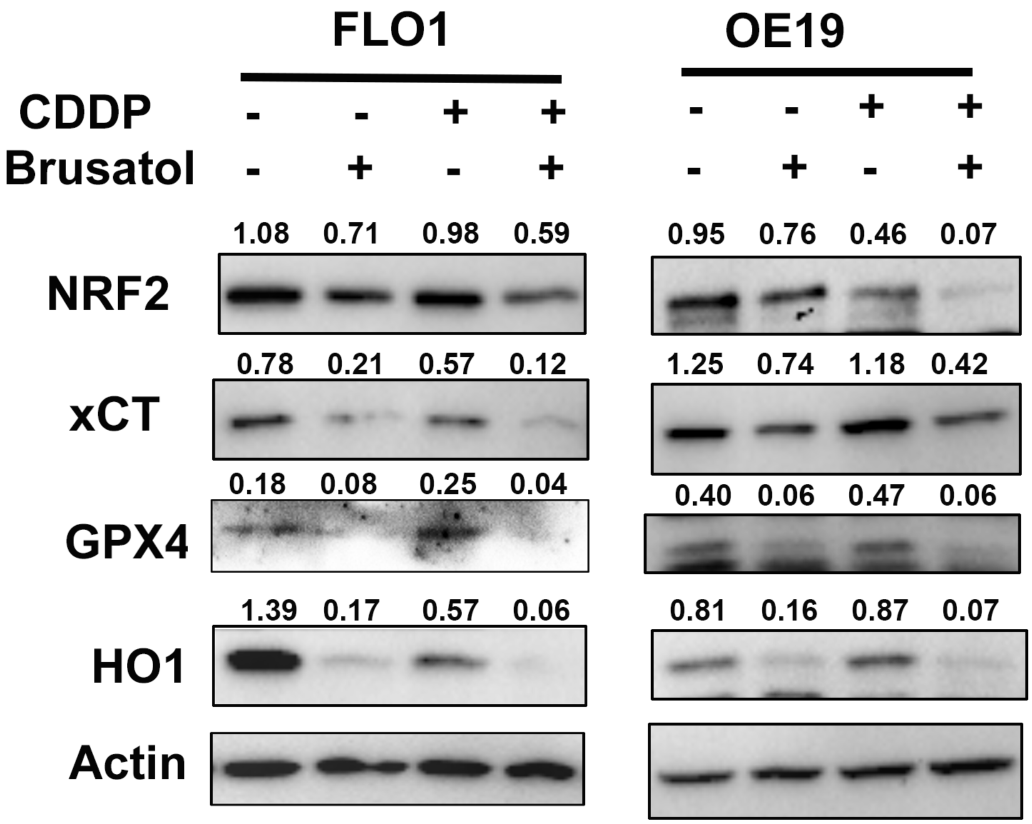

Supplementary Figure S6. Brusatol or combination of Brusatol and CDDP induced ferroptosis in FLO1 and OE19 cells*. Western blotting assay shows reduced levels of xCT and GPX4, two ferroptosis markers, in Brusatol or combination treated cells, together with down regulation of HO1, a known NRF2 target gene. The numbers above each band indicate the relative band intensity as normalized to the intensity of the loading control, Actin.*

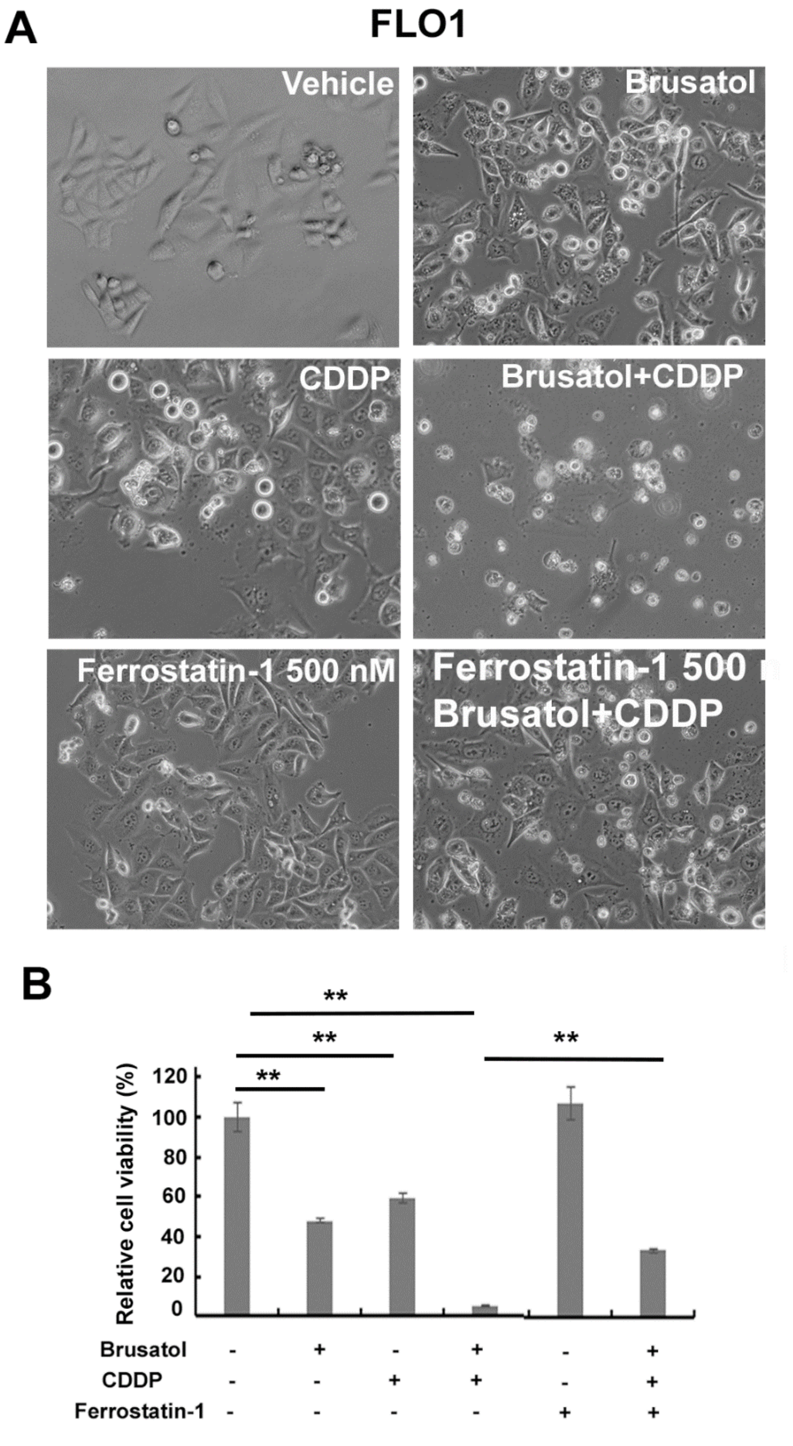

Supplementary Figure S7. Ferrostatin 1 protected cells from cell death*. FLO1 cells were added with Brusatol or CDDP alone or combination or Ferrostatin 1 (500 nM) together with Brusatol and CDDP for 72h.* ***A****, Bright images under an inverted microscope.* ***B****, Summary of ATPglo assay shows relative cell viability after 3 days above treatments. **, p <0.01.*

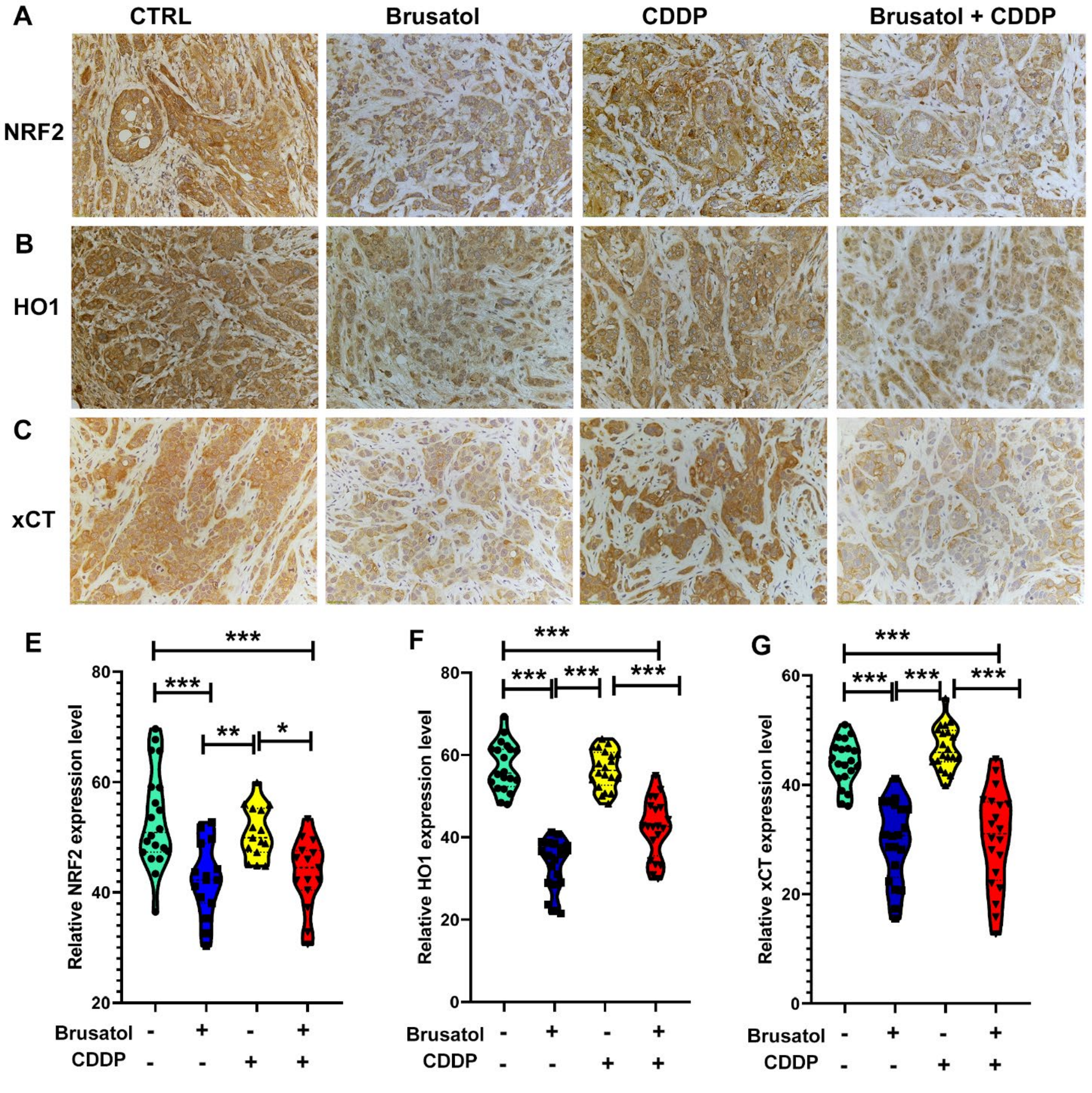

Supplementary Figure S8. IHC staining of NRF2, HO1, xCT in xenografting tumor tissues. ***A***, *representative images of NRF2,* ***B***, *representative images of HO1,* ***C***, *representative images of xCT.* ***E-G,*** *quantification of IHC staining intensity using ImageJ software and statistical analysis was performed using Prism software,* ***E*** *for NRF2,* ***F*** *for HO1 and* ***G*** *for xCT. *, p<0.05; **, p<0.01; ***, p<0.001.*

Supplementary Table S1: Primers for real time PCR

Gene name	Forward primer sequence	Reverse primer sequence
NFE2L2	ACACGGTCCACAGCTCATC	TCTTGCCTCCAAAGTATGTCAA
HO1	AACTTTCAGAAGGGCCAGGT	CTTGTTGCGCTCAATCTCCT
GR	GTGGTGGAGAGCCACAAGC	ACCCTCACAACTTGGAAAGC
NQO1	CAGCTCACCGAGAGCCTAGT	CAGCCTCCTTCATGGCATAG
GSTM1	AGGACTTCATCTCCCGCTTT	AGGCTGAGTATGGGCTCCTC
GSTM2	CCTTCCCAAACCTGAAGGA	TTCAAGGCCCTACTTGTTGC
GSTM3	GCTCCTGGAGTTCACGGATA	GCATTGCTCTGGGTGATCTT
GSTO1	GGACGCGTCTAGTCCTGAAG	CAGGTGATGGCAGACTCGTAG
GPX3	GCC GGG GAC AAG AGA AGT	GAG GAC GTA TTT GCC AGC AT
GPX4	GCCTTTGCCGCCTACTGA	TAACCATGTGCCCGTCGAT
